# Loss of NF-κB p50 function synergistically augments microglial priming in the middle-aged brain

**DOI:** 10.1186/s12974-019-1446-z

**Published:** 2019-03-12

**Authors:** Thomas Taetzsch, Savannah Benusa, Shannon Levesque, Christen L. Mumaw, Michelle L. Block

**Affiliations:** 1 Department of Anatomy and Neurobiology, Virginia Commonwealth University Medical Campus, Richmond, VA 23298 USA; 2 Department of Anatomy and Cell Biology, The Stark Neuroscience Research Institute, Indiana University School of Medicine, Indianapolis, IN 46202 USA

**Keywords:** Microglia, Aging, NF-κB, Priming

## Abstract

**Background:**

While NF-κB p50 function is impaired in central nervous system disease, aging in non-CNS tissues, and response to reactive oxygen species, the role of NF-κB p50 in aging-associated microglial pro-inflammatory priming is poorly understood.

**Methods:**

Male NF-κB p50^+/+^ and NF-κB p50^−/−^ mice at three different ages (1.5–3.0 month old, 8.0–11.0 month old, and 16.0–18.0 month old) were treated with LPS (5 mg/kg, IP) to trigger peripheral inflammation, where circulating cytokines, neuroinflammation, microglia morphology, and NF-κB p50/p65 function in brain tissue were determined 3 h later.

**Results:**

Peripheral LPS injection in 9-month-old C57BL/6 mice resulted in lower NF-κB p50 DNA binding of nuclear extracts from the whole brain, when compared to 3-week-old C57BL/6 mice, revealing differences in LPS-induced NF-κB p50 activity in the brain across the mouse lifespan. To examine the consequences of loss NF-κB p50 function with aging, NF-κB p50^+/+^ and NF-κB p50^−/−^ mice of three different age groups (1.5–3.0 month old, 8.0–11.0 month old, and 16.0–18.0 month old) were injected with LPS (5 mg/kg, IP). NF-κB p50^−/−^ mice showed markedly elevated circulating, midbrain, and microglial TNFα when compared to NF-κB p50^+/+^ mice at all ages. Notably, the 16.0–18.0-month-old (middle aged) NF-κB p50^−/−^ mice exhibited synergistically augmented LPS-induced serum and midbrain TNFα when compared to the younger (1.5–3.0 month old, young adult) NF-κB p50^−/−^ mice. The 16.0–18.0-month-old LPS-treated NF-κB p50^−/−^ mice also had the highest midbrain IL-1β expression, largest number of microglia with changes in morphology, and greatest elevation of pro-inflammatory factors in isolated adult microglia. Interestingly, aging NF-κB p50^−/−^ mice exhibited decreased brain NF-κB p65 expression and activity.

**Conclusions:**

These findings support that loss of NF-κB p50 function and aging in middle-aged mice may interact to excessively augment peripheral/microglial pro-inflammatory responses and point to a novel neuroinflammation signaling mechanism independent the NF-κB p50/p65 transcription factor in this process.

## Background

As resident sentinels surveying the central nervous system (CNS) environment [1], electricians regulating synaptic communication [2], and innate immune cells of the brain [3], microglia are essential to CNS maintenance and health [4]. Microglia respond to a host of stimuli including cellular damage, environmental toxins, and pathogens [5] with a carefully orchestrated activation response. Neuropathology and CNS disease is believed to occur when the microglial pro-inflammatory response is exacerbated and unregulated [5–7], which is linked to microglial priming [8]. Microglia have long been implicated in late-onset neurodegenerative diseases [9] that are closely related to aging [10], such as Parkinson’s disease.

Current theory holds that a phenotypic shift in microglia towards a more reactive primed state occurs as the brain ages [11–14]. While there are some conflicting reports in the literature [15], aged microglia challenged with an innate immune stimulus, such as peripheral LPS, are generally reported to exhibit a significantly more pronounced pro-inflammatory response [16], which is referred to as priming. In fact, even without a distinct innate immune trigger, the aged brain is reported to display heightened basal levels of pro-inflammatory cytokines, such as TNFα, IL-1β, and IL-6 [17–19] along with lower levels of alternative activation and anti-inflammatory factors, including IL-4 [20], IL-4 receptor [21], IL-10 [19], fractalkine [22, 23], and CD200 [24]. The mechanisms underlying the changing neuroinflammation responses across the lifespan are largely undefined.

While the causes driving the enhanced sensitivity of aging microglia to proinflammatory stimuli are poorly understood, age-related microglial priming is predicted to be an interaction between the microglial responses to increasingly deleterious changes in the CNS environment [25] and intrinsic microglial characteristics associated with aging [12]. For example, several factors are implicated in the process of age-induced microglial priming, including upregulation of innate immune receptors on microglia responsible for the detection of pro-inflammatory stimuli [12, 25], enhanced peripheral inflammation/circulating cytokine production that transfers to the brain to impact neuroinflammation [26], and elevated states of oxidative stress in the aged brain [27–29]. However, the intrinsic signaling mechanisms that shift microglia into a primed state as the brain ages remain unclear.

Previous reports point to NF-κB p50 as an essential pleiotropic regulator of the pro-inflammatory response [30], where loss of NF-κB p50 function due to reactive oxygen species (ROS) [7] or genetic deletion resulted in dysregulated microglial activation culminating in chronically elevated TNFα production in the brain [7, 31]. While little is currently known about NF-κB p50 function in the aged brain, loss of NF-κB p50 function has been linked to CNS disease, where patients with Lewy Body Dementia have been documented to express less NF-κB p50 in the substantia nigra [32]. Mechanistically, the NF-κB family of transcription factors are essential regulators of the pro-inflammatory response [33, 34], where the NF-κB p50/p65 heterodimer is a prototypical pro-inflammatory transcription factor linked to the initiation of the expression of pro-inflammatory genes [35, 36]. While less intuitive, the NF-κB p50/p50 homodimer is a repressor of the pro-inflammatory response that is both constitutively bound and activated with delayed kinetics to regulate the pro-inflammatory response [35, 36]. Mice that lack functional NF-κB p50 are more sensitive to neurotoxins, display memory deficits [37], show an aberrant microglia-mediated inflammatory response in a rodent Alzheimer’s disease model [31], exhibit a dysfunctional kinetic response of the microglial pro-inflammatory response, and are vulnerable to chronic neuroinflammation [7]. Global knockout of *NFκB1*, the gene that encodes NF-κB p50, causes a loss of NF-κB p50 function in all cells which can obscure the assessment of immune-mediated neurotoxicity, depending on the model, where NF-κB p50^−/−^ mice are protected from ischemia-induced neuronal apoptosis [38]. Importantly, *NFκB1* knockout mice demonstrate chronic low-grade inflammation across their lifespan and accumulate telomere dysfunction and prematurely senescent cells in the periphery, supporting that loss of NF-κB p50 function may accelerate aging [39–42]. Further, in peripheral tissues, NF-κB p50 function is reported to decline with age [40, 42]. Thus, while NF-κB p50 is associated with aging/cellular senescence, regulates the microglial pro-inflammatory response, and has CNS effects, the role of NF-κB p50 in microglial aging is unknown.

Here, to begin to explore the role of NF-κB p50 in microglial pro-inflammatory priming with age, we addressed (1) the impact of aging on NF-κB p50 function in the brain and (2) the effect of loss of NF-κB p50 function on LPS-induced circulating cytokines, neuroinflammation, and the production of pro-inflammatory factors in microglia in the aging brain. We demonstrate that the brain NF-κB p50 response to peripheral inflammation changes across the lifespan and that microglial priming and neuroinflammation in middle-aged mice is synergistically amplified by the loss of NF-κB p50 function and aging.

## Methods

### Reagents

Lipopolysaccharide (0111,B4, lot 050 M4100) was obtained from Sigma-Aldrich (St. Louis, MO). Cell culture reagents were purchased from Invitrogen (Carlsbad, CA) and Corning (Corning, NY). Phosphatase inhibitor and HALT protease inhibitor were acquired from Thermo Fisher Scientific (Rockford, IL). All other reagents were purchased from Sigma-Aldrich Chemical Company (St. Louis, MO).

### Animals

NF-κB p50-deficient mice (B6.Cg-*Nfkb1* ^tm1Bal^/J, NF-κB p50^−/−^) and C57BL/6J (NF-κB p50^+/+^) mice were purchased from Jackson Laboratories (Bar Harbor, Maine) and maintained in a strict pathogen-free environment. NF-κB p50^−/−^ mice have a targeted mutation of exon 6 in the Nfkb1 gene that results in non-functional NF-κB p105 and NF-κB p50 proteins due to a truncated peptide that prevents dimerization with NF- κB subunits and DNA binding [43]. The NF-κB p50^−/−^ mutation is maintained in the C57BL/6J background; therefore, C57BL/6J (NF-κB p50^+/+^) mice were used as controls. National Institutes of Health guidelines for breeding, housing, and experimental use of the animals were strictly followed.

### In vivo LPS treatment

Male NF-κB p50^+/+^ and NF-κB p50^−/−^ mice of three different age groups (1.5–3.0 month old, 8.0–11.0 month old, and 16.0–18.0 month old) received a single intraperitoneal (IP) injection of LPS (5 mg/kg) or equivalent volume of vehicle (0.9% saline). The administration of the single LPS (5 mg/kg, IP) injection is a murine Parkinson’s disease model of chronic neuroinflammation that persists across the lifespan of the mouse and culminates in delayed and progressive DA neuron loss in the substantia nigra that begins at 7 months after the injection [29, 44]. Based on our previous work, this LPS dose causes peripheral inflammation that rapidly transfers to the brain causing microglial activation in the substantia nigra in young adult NF-κB p50^+/+^ and NF-κB p50^−/−^ mice, that is evident at 3 h after injection [7]. As such, similar to our prior work [7], circulating cytokines, microglial activation, and midbrain (which contains the substantia nigra)/whole brain neuroinflammation were assessed at 3 h post-injection, where protein, mRNA, or morphological analyses were performed on serum and brain tissue following collection.

### Immunohistochemistry

Immunohistochemistry was performed on 40-μm coronal sections of the midbrain region as described previously [7]. Briefly, free-floating brain slices were stained with anti-tyrosine hydroxylase (TH, 1:1000) rabbit polyclonal antibody (Millipore Billerica, MA) to visualize dopaminergic neurons to confirm that sectioned samples across mice were in the same frame of the substantia nigra pars compacta (SNpc) and a polyclonal rabbit anti-ionized calcium-binding adaptor molecule-1 (Iba1, 1:1000) antibody (Wako, Richmond, VA) was used to identify microglia as previously described. Images were captured with an Olympus BX51 microscope (Olympus America, Center Valley, PA) [7].

### Nuclear protein extraction and NF-κB p50 and p65 DNA binding ELISA

A commercially available Nuclear Extract Kit (Active Motif, Carlsbad, CA) was used to collect nuclear protein from whole brain tissue, as we have previously reported [7]. The TransAM NFκB ELISA (Active Motif, Carlsbad, CA) was used to assess DNA binding based on the ability of nuclear protein to bind a generic NF-κB DNA consensus site (5′-GGGACTTTCC-3′) immobilized on a 96 well plate according to manufacturer’s instructions.

### NF-κB p65 Western blot

Ten micrograms of nuclear extract were electrophoresed on a 12% SDS-PAGE gel. Samples were transferred to nitrocellulose membranes by semi-dry transfer, blocked with 5% nonfat milk for 1 h at 24 °C, followed by incubation overnight with mouse anti-GAPDH (Millipore, Billerica, MA, 1:1000) or rabbit anti-NF-κB p65 (SC-372, Santa Cruz Biotechnology, Dallas, TX, 1:1000) antibodies at 4 °C. Blots were then incubated with horseradish peroxidase-linked mouse anti-rabbit (1:2500) or goat anti-mouse (1:5000) for 1 h (24 °C), and ECL Plus reagents (Amersham Biosciences Inc., Piscataway, NJ) were used as a detection system. Band density was quantitated with ImageJ [34] and analyzed as a ratio of GAPDH and NF-κB p65. Results are reported as a percent increase from control.

### Quantitative reverse transcriptase polymerase chain reaction

Total RNA was extracted from the mouse brain or from isolated microglia using Trizol (Invitrogen Life Technologies, Grand Island, NY) according to manufacturer’s instructions. The RNA was treated with Ambion DNase I (Invitrogen Life Technologies, Grand Island, NY) which was subsequently removed with Qiagen RNeasy RNA Cleanup Kit (Qiagen, Germantown, MD). Reverse transcription of RNA (0.3–1.0 μg/sample) was performed with iScript Reverse Transcription Supermix (BioRad, Hercules, CA) according to manufacturer’s instructions. SsoFast Evagreen Supermix (BioRad, Hercules, CA) and 500 nM forward and reverse primers were used to carry out quantitative PCR on a CFX96 (BioRad, Hercules, CA) real-time PCR detection system, per manufacturer’s instructions. Cycling parameters were 1 cycle at 95 °C for 5 min and 40 cycles of 95 °C (5 s) and 56 °C (5 s) followed by a melt curve measurement consisting of 5 s 0.5°C incremental increases from 65 °C to 95 °C. Primer sequences used in this study are listed in Table 1.

**Table 1:** Primer Sequences

Gene	Forward primer	Reverse primer
COX-2	5′-TTGCTGGCCGGGTTGCTGG-3′	5′-CAGGGAGAAGCGTTTGCGGT-3′
GAPDH	5′-AACTTTGGCATTGTGGAAGG-3′	5′-ATCCACAGTCTTCTGGGTGG-3′
IL-1β	5′-TGAAGAAGAGCCCATCCTCTGTGA-3′	5′-GGTCCGACAGCACGAGGCTT-3′
iNOS	5′-TCCAGAATCCCTGGACAAGCTGC-3′	5′-TGCAAGTGAAATCCGATGTGGCCT-3′
TNFα	5′-GCCCACGTCGTAGCAAACCACC-3′	5′-CCCATCGGCTGGCACCACTA-3′

*COX-2* cyclooxygenase 2, *GAPDH* glyceraldehyde 3-phosphate dehydrogenase, *IL-1β* Interleukin 1β, *iNOS* inducible nitric oxide synthase, *TNFα* tumor necrosis factor α

### Adult microglia isolation

Adult microglia isolation was performed as previously described [7, 45]. Briefly, mice were anesthetized at 3 h post-injection and perfused with 50 mL cold PBS and microglia were isolated using the Miltenyi Neural Tissue Dissociation Kit (P), (Miltenyi Biotec, San Diego, CA) according to manufacturer’s instructions, which results in 87.0% pure microglia/CD11b positive cells [7].

### Tumor necrosis factor α ELISA

The TNFα concentrations in sera and cell culture media were measured with a commercial ELISA kit from R&D Systems (Minneapolis, MN), as previously reported [7, 44].

### Stereology: assessment of microglial activation

The fractionator method of unbiased stereology was utilized to evaluate microglial activation in the SNpc, as previously described [7, 46]. Briefly, TH-stained coronal sections located at − 3.14, − 3.26, and − 3.38 mm bregma were used to delineate the SNpc. A 40× objective was used to score stages of the changes in microglial morphology of IBA-1 stained microglia within the SNpc. Morphological parameters were used to identify four distinct morphology classifications (0–3) as previously described [7, 46]. Samples were counted in a blind manner by two individuals using an Olympus BX51 microscope (Center Valley, PA) and newCAST software (Visiopharm, Hoersholm, Denmark). Conclusions were drawn only when differences in counts were less than 12% between individuals.

### Statistical analysis

One- or two-way analysis of variance and Bonferroni’s post-hoc analysis were used for statistical evaluation. Treatment groups were expressed as the mean ± SEM. An independent *t* test was used in cases where only two means could be compared. A value of *P* < 0.05 was considered statistically significant.

## Results

### NF-κB p50 activity in response to LPS changes across the lifespan

**Fig. 1:**
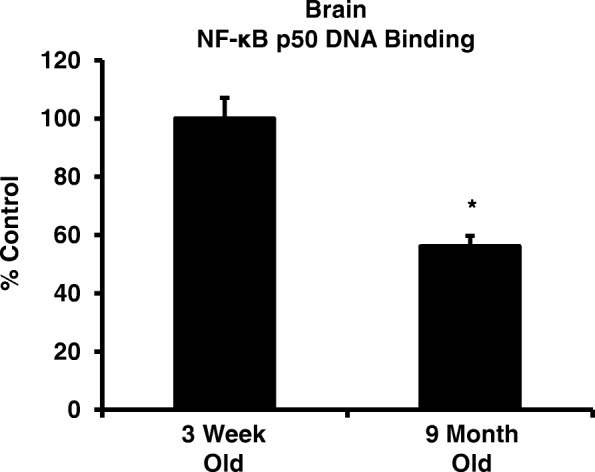
LPS-induced NF-κB p50 DNA binding changes across the mouse lifespan. NF-κB p50 activity was assessed by determining NF-κB p50 DNA binding in whole brain nuclear extract from 9-month-old and 3-week-old C57BL/6 mice injected with LPS (5 mg/kg, IP). Whole brain nuclear extract from the older mice injected with LPS showed reduced DNA binding when compared to the younger mice. Values are reported as mean percent of 3 week saline control ± SEM. An asterisk indicates significant difference (*P* < 0.05) from control. *n* = 4

While decreased NF-κB p50 expression has been linked to CNS disease [32], how aging affects NF-κB p50 function in the brain is poorly understood. To test the possibility of age-specific effects on NF-κB p50 activity, whole brain nuclear extract was collected from young (3-week-old) and older (9-month-old) LPS-treated C57BL/6 mice and NF-κB p50 DNA binding was assessed. Peripherally administered LPS (IP) causes an inflammatory response characterized by elevated circulating pro-inflammatory cytokines that transfers to the brain resulting in microglial activation, which has been implicated in neuropathology [44, 47, 48]. The 9-month-old mice treated with LPS displayed significantly lower levels of NF-κB p50 DNA binding when compared to young LPS-treated mice (Fig. 1, *P* < 0.05), supporting that NF-κB p50 activity in response to pro-inflammatory triggers changes across the lifespan. The consequences of this change in NF κB activity across the lifespan, particularly a decrease in NF-κB p50 function, are poorly understood.

### Aging and loss of NF-κB p50 function synergistically interact to amplify serum and brain TNFα

**Fig. 2:**
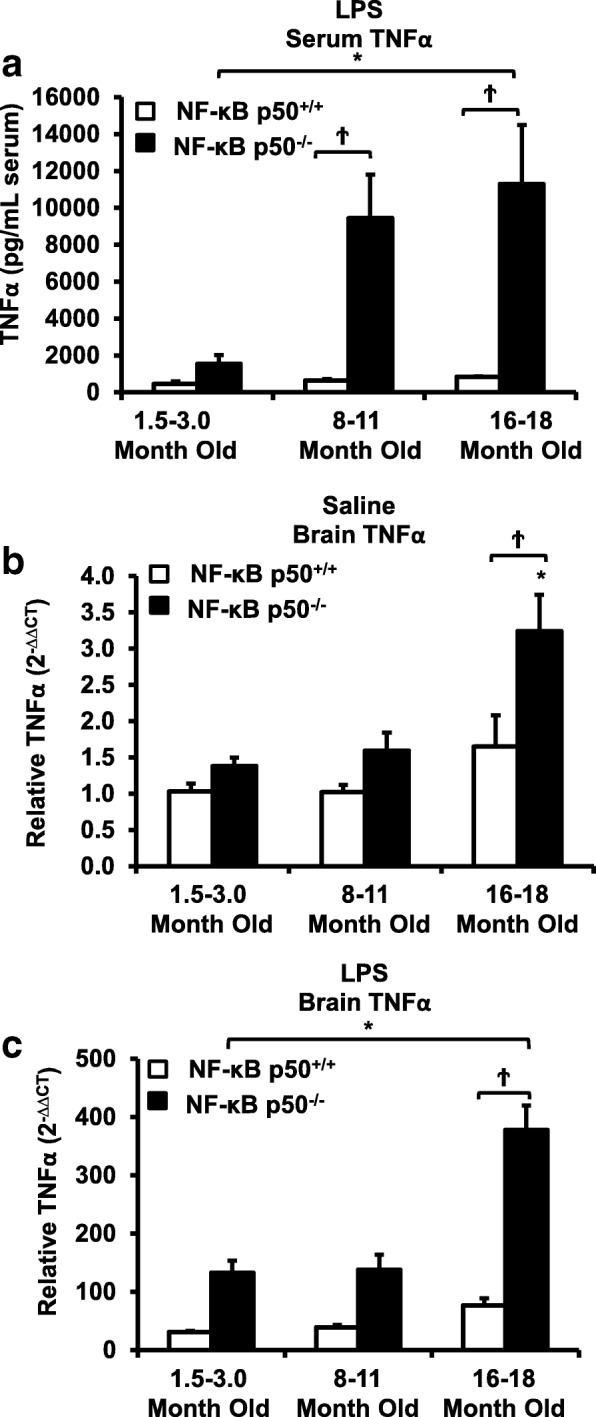
Loss of NF-κB p50 function and aging synergistically interact to augment serum and brain TNFα in middle-aged mice. NF-κB p50^+/+^ and NF-κB p50^−/−^ mice from three different age groups (1.5–3.0 month old, 8.0–11.0 month old, and 16.0–18.0 month old) were injected with saline or LPS (5 mg/kg, IP) to determine age-related effects of loss of NF-κB p50 function on peripheral and neuro-inflammation at 3 h post-injection. **a** Circulating TNFα protein was measured by ELISA. Values are reported as mean expression ± SEM. Midbrain TNFα mRNA levels were assessed following **b** saline or **c** LPS treatment by quantitative RT-PCR. Values were normalized to GAPDH using the 2^−ΔΔCT^ method and are reported as mean percent of saline control ± SEM. An asterisk indicates significant difference (*P* < 0.05) from the 1.5–3.0-month-old group and a dagger indicates a significant difference between mouse strains. *n* = 3–7

To address the effects of loss of NF-κB p50 function on age-related increases in peripheral inflammation and microglial priming, NF-κB p50^+/+^ and NF-κB p50^−/−^ mice in three age groups (1.5–3.0 month old, 8.0–11.0 month old, and 16.0–18.0 month old) were treated with LPS and TNFα levels in serum and midbrain were evaluated at 3 h post-injection. There were no significant differences in baseline serum TNFα in any group tested (data not shown). In response to LPS, serum TNFα levels in the 8.0–11.0 and 16.0–18.0 month old age bracket were strikingly elevated in NF-κB p50^−/−^ mice (Fig. 2a, *P* < 0.05), but only the 16.0–18.0-month-old NF-κB p50^−/−^ mice were significantly higher than the genotype augmented TNFα levels in 1.5–3.0-month-old NF-κB p50^−/−^ mice (Fig. 2a, *P* < 0.05). The midbrain exhibited a unique pattern, where there was a trend for increased basal midbrain TNFα levels with saline in 16–18-month-oldNF-κB p50^−/−^ mice (Fig. 2b, P =.099), suggesting that aging and loss of NF-κB p50 function may mildly elevate basal levels of brain cytokines. While the brain TNFα response to LPS was similar in the 1.5–3.0 month old and 8.0–11.0-month-old NF-κB p50^−/−^ mice, the 16–18-month-old NF-κB p50^−/−^ mice exhibited synergistically higher levels of TNFα, when compared to 1.5–3.0-month-old NF-κB p50^−/−^ mice (Fig. 2c, *P* < 0.05). Together, these results support that aging and loss of NF-κB p50 function interact to augment inflammation in both the periphery and brain and that the CNS vulnerability to transfer of the synergistic peripheral effects may elevate in middle age.

### Aging and loss of NF-κB p50 function synergistically interact to amplify midbrain IL-1β

**Fig. 3:**
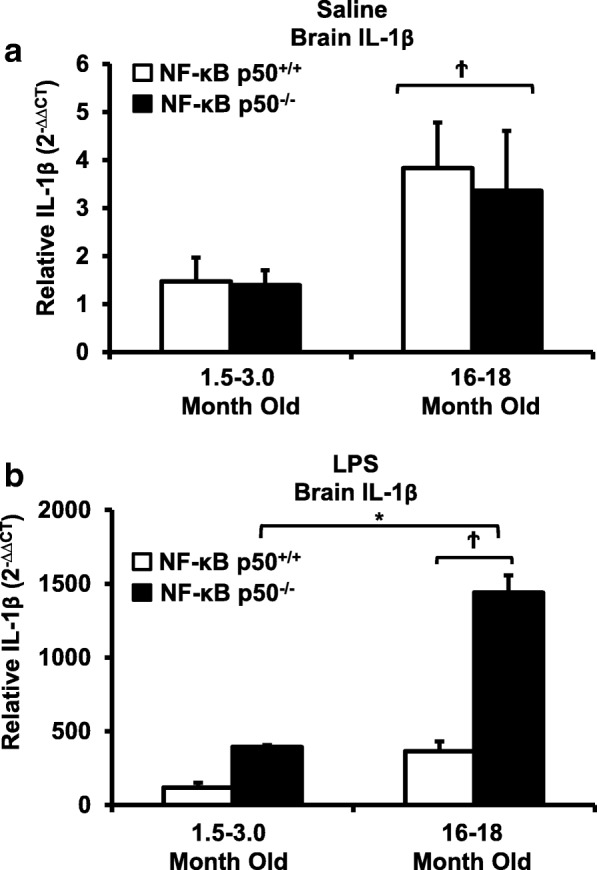
Loss of NF-κB p50 function and aging synergistically interact to augment midbrain IL-1β in middle-aged mice. Young adult (1.5–3.0 month old) and middle-aged (16.0–18.0 month old) NF-κB p50^+/+^ and NF-κB p50^−/−^ mice were treated with **a** saline or **b** LPS (5 mg/kg, IP), and brain tissue was collected at 3 h post-injection. IL-1β expression in the brain was evaluated by quantitative RT-PCR. Values are normalized to GAPDH using the 2^−ΔΔCT^ method and are reported as mean percent of NF-κB p50^+/+^ young adult saline control ± SEM. An asterisk indicates significant difference (*P* < 0.05) from the 1.5–3.0-month old group and a dagger indicates a significant difference between mouse strains. *n* = 3

To further characterize the effects of aging on NF-κB p50-mediated priming of neuroinflammation, we assessed levels of additional pro-inflammatory markers in LPS-injected 1.5–3.0 (young adult) and 16–18 (middle aged)-month-old NF-κB p50^+/+^ and NF-κB p50^−/−^ mice. An age-related increase in basal IL-1β expression was observed in saline-treated mice, regardless of genotype (Fig. 3a, *P* < 0.05). Peripheral LPS injection resulted in a significant increase in brain IL-1β expression in middle-aged NF-κB p50^−/−^ mice versus both middle-aged NF-κB p50^+/+^ mice and young adult NF-κB p50^−/−^ mice (Fig. 3b, P < 0.05). Consistent with our previous reports documenting no differences in LPS-induced 3-h COX2 and iNOS gene expression in young adult NF-κB p50^−/−^ mice [7], genotype and age-related differences in brain iNOS and COX2 expression were not observed (data not shown). These findings indicate that aging and loss of NF-κB p50 function interact to regulate IL-1β in the midbrain in middle-aged mice.

### Aging and NF-κB p50 affects microglia morphology and overall number in the substantia nigra

**Fig. 4:**
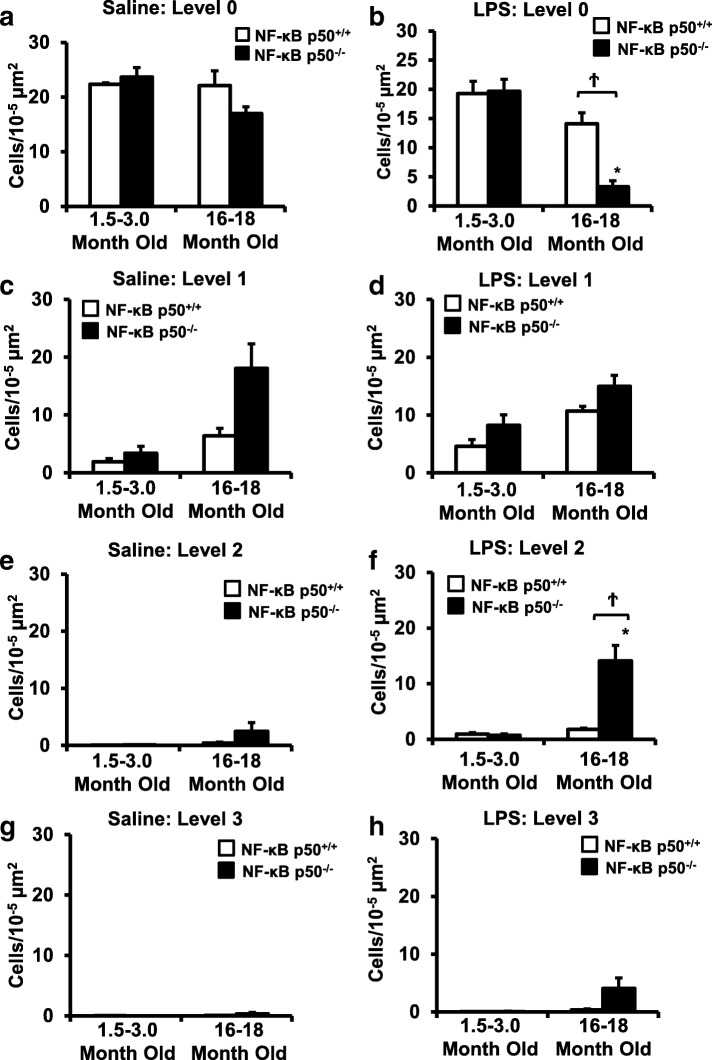
Aging NF-κB p50^−/−^ mice have enhanced activated microglia morphology in response to peripheral LPS injection. Young adult (1.5–3.0 month old) and middle-aged (16.0–18.0 month old) NF-κB p50^+/+^ and NF-κB p50^−/−^ mice were injected with saline or LPS (5 mg/kg, IP) to evaluate loss of NF-κB p50 function on microglia morphology in vivo. IBA1 stained microglia within the substantia nigra pars compacta (in the midbrain) were categorized into stages of activation ranging from resting (stage 0) to highly activated (stage 3). The relative number of microglia at 3 h post-injection within stage 0 (**a**, **b**), stage 1(**c**, **d**), stage 2(**e**, **f**), and **g** & **h** stage 3(**g**, **h**) was quantified by the fractionator method. Values are reported as mean cells/μm^2^ ± SEM of 3 coronal sections (40 μm) per animal. An asterisk indicates significant difference (*P* < 0.05) from control and a dagger indicates a difference between mouse strains. *n* = 3

**Fig. 5:**
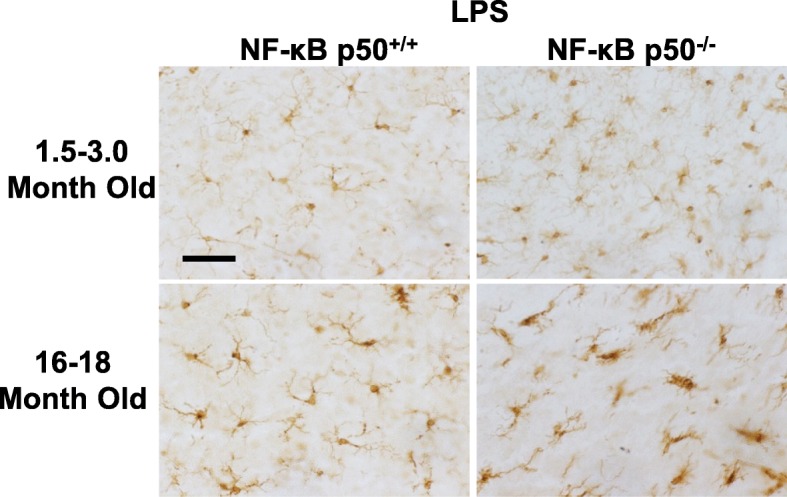
Representative images demonstrating augmented changes in microglia morphology in middle-aged NF-κB p50^−/−^ mice. Young adult (1.5–3.0 month old) and middle-aged (16.0–18.0 month old) NF-κB p50^+/+^ and NF-κB p50^−/−^ mice were injected with saline or LPS (5 mg/kg, IP) to evaluate loss of NF-κB p50 function on microglia morphology in vivo. Three coronal sections (40 μm) per animal containing the substantia nigra pars compacta were stained with IBA1. Representative images are shown from the substantia nigra pars compacta at × 40 and the scale bar depicts 50 μM. *n* = 3

To examine the age-related effects of loss of NF-κB p50 function on microglia, an analysis of microglia morphology in the substantia nigra pars compacta (SNpc) of young adult (1.5–3 months) and middle-aged (16–18 months) NF-κB p50^+/+^ and NF-κB p50^−/−^ mice was performed. While no differences in the number of resting (Level 0) microglia were observed in saline-treated mice (Fig. 4a), a significant decrease in the number of resting (Level 0) microglia was observed in the SNpc of LPS-treated middle-aged NF-κB p50^−/−^ mice as compared to LPS-treated middle-aged NF-κB p50^+/+^ mice and young adult NF-κB p50^−/−^ mice (Figs. 4b and 5, *P* < 0.05). Middle-aged saline-treated NF-κB p50^−/−^ mice displayed a modest increase in basal levels of microglia with mild morphological changes (Level 1) as compared to middle-aged NF-κB p50^+/+^ mice; however this difference was not significant (Fig. 4c, *P* = 0.06). Among LPS-treated mice, numbers of microglia classified with a level 1 morphology were similar across age and genotype (Figs. 4d and 5). Middle-aged LPS-injected NF-κB p50^−/−^ mice showed significantly increased numbers of microglia exhibiting the level 2 morphology as compared to aged LPS-injected NF-κB p50^+/+^ mice (Figs. 4f and 5, *P* < 0.05). LPS-injected middle-aged NF-κB p50^−/−^ mice showed an increase in numbers of microglia with the level 3 morphology, a morphology not observed in NF-κB p50^+/+^ mice. However, significant differences were not observed (Figs. 4h and 5, *P* = .078). Few microglia classified as stage 2 or stage 3 morphology were observed in the SNpc of saline-treated mice (Fig. 4e and g). Additionally, both NF-κB p50^+/+^ and NF-κB p50^−/−^ mice displayed an age-related increase in overall microglia number (stages 0–3) in the SNpc with the largest age-related increases in microglia number occurring in NF-κB p50^−/−^ mice (Table 2, *P* < 0.05). These findings demonstrate an overall increase in microglia number with age as well as an age-related increase in changes in morphology in response to LPS in the absence of NF-κB p50, which is characterized by lower numbers of quiescent microglia and elevated numbers of microglia displaying hypertrophic morphology.

**Table 2:** Microglia number. Cell counts in young adult and middle-aged NF-κB p50^+/+^ and NF-κB p50^−/−^ mice

Group	Mean cells/10^− 5^ μm^2^	SEM	*P* value
Young p50^+/+^	6.145	0.134	
Middle-aged p50^+/+^	6.985*	0.294	.026
Young p50^−/−^	6.973	0.346	
Middle-aged p50^−/−^	9.283*	0.587	.007

The relative number of microglia counted in all stages of morphology (0–3) in the SNpc was quantified by the fractionator method. Values are reported as mean cells/μm^2^ of 3 coronal sections (40 μm) per animal. *Significant difference (*P* < 0.05) from young genotype-matched mice. *n* = 3

### Aging and loss of NF-κB p50 synergistically amplify IL-1β and TNFα in microglia

**Fig. 6:**
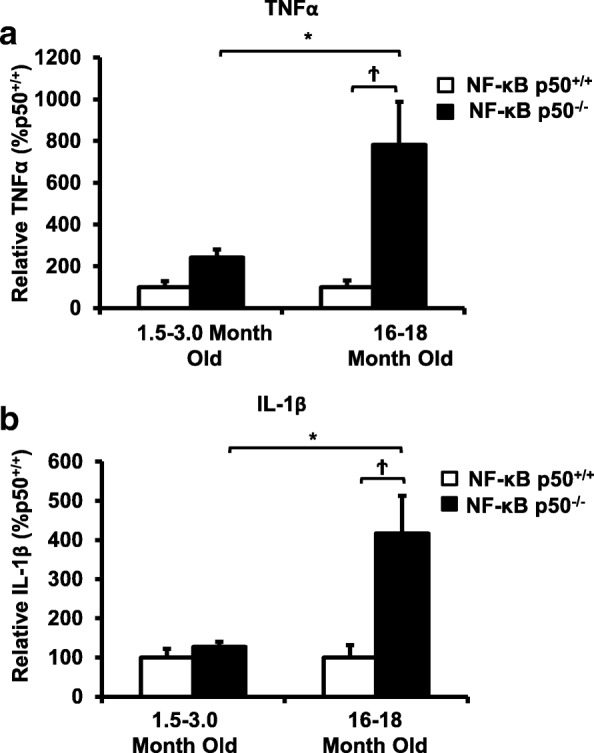
Loss of NF-κB p50 function and aging synergistically interact to amplify TNFα and IL-1β in isolated microglia. Young adult (1.5–3.0 month old) and middle-aged (16.0–18.0 month old) NF-κB p50^+/+^ and NF-κB p50^−/−^ mice were injected with LPS (5 mg/kg, IP), and isolation of microglia from the whole brain with CD11b microbeads was performed at 3 h post-injection. Differences in **a** TNFα and **b** IL-1β were evaluated by quantitative RT-PCR. Values are normalized to GAPDH using the 2^−ΔΔCT^ method and are reported as mean percent of NF-κB p50^+/+^ young adult saline control ± SEM. An asterisk indicates significant difference (*P* < 0.05) from the 1.5–3-month group and a dagger indicates a difference between mouse strains. *n* = 3

To discern the age-related effects of loss of NF-κB p50 function specifically in microglia, young adult and middle-aged mice of both genotypes were injected with LPS and gene expression was evaluated in CD11b microbead-isolated microglia at 3 h post-injection. Microglia from saline-treated mice exhibited no significant differences in basal TNFα nor IL-1β gene expression, except that young adult NF-κB p50^−/−^ mice had higher basal levels of TNFα expression (Tables 3 and 4, *P* < 0.05). Microglia isolated from the brains of middle-aged LPS-injected NF-κB p50^−/−^ mice showed significantly elevated TNFα (Fig. 6a, *P* < 0.05) and IL-1β (Fig. 6b, *P* < 0.05) expression as compared to young LPS-treated NF-κB p50^+/+^ mice, supporting that loss NF-κB p50 function amplifies microglial sensitivity to pro-inflammatory stimuli in the aging brain.

**Table 3:** TNFα mRNA expression in microglia isolated from saline-treated control mice

Group	TNFα expression	SEM	*P* value
Young p50^+/+^	100.00	12.36	
Young p50^−/−^	242.12*	42.35	< 0.05
Middle-aged p50^+/+^	100.00	6.17	
Middle-aged p50^−/−^	175.19	42.76	

Young adult (1.5–3.0 month old) and middle-aged (16.0–18.0 month old) NF-κB p50^+/+^ and NF-κB p50^−/−^ mice were injected with saline, and isolation of microglia from the whole brain with CD11b microbeads was performed at 3 h post-injection. TNFα mRNA levels were evaluated by quantitative RT-PCR. Values are normalized to GAPDH using the 2^−ΔΔCT^ method. *Significant difference (*P* < 0.05) between mouse strains. *n* = 3

**Table 4:** IL-1β mRNA expression in microglia isolated from saline-treated control mice

Group	IL-1β	SEM	*P* value
Young p50^+/+^	100.00	38.00	
Young p50^−/−^	95.93	24.00	
Middle-aged p50^+/+^	100.00	12.30	
Middle-aged p50^−/−^	120.00	57.40	

Young adult (1.5–3.0 month old) and middle-aged (16.0–18.0 month old) NF-κB p50^+/+^ and NF-κB p50^−/−^ mice were injected with saline, and isolation of microglia from the whole brain with CD11b microbeads was performed at 3 h post-injection. IL-1β mRNA levels were evaluated by quantitative RT-PCR. Values are normalized to GAPDH using the 2^-ΔΔCT^ method. *Significant difference (*P* < 0.05) between mouse strains. *n* = 3

### NF-κB p65 activation and expression decreases in middle-aged mice

**Fig. 7:**
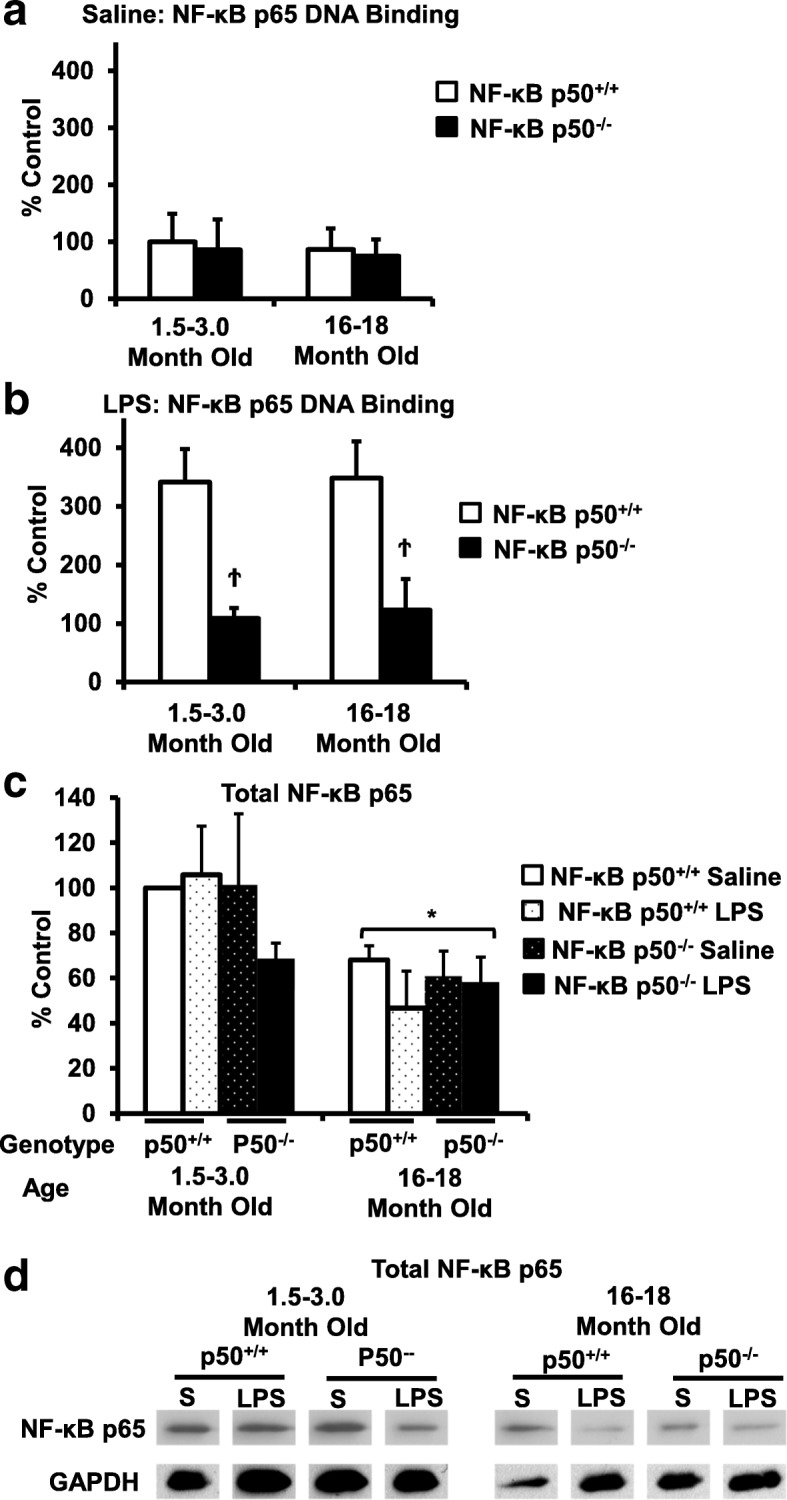
NF-κB p65 DNA expression and activity in aging and NF-κBp50^−/−^ mice. Young adult (1.5–3.0 month old) and middle-aged (16.0–18.0 month old) NF-κB p50^+/+^ and NF-κB p50^−/−^ mice were injected with saline or LPS (5 mg/kg, IP). Nuclear extract collected from whole brain tissue at 3 h following **a** saline or **b** LPS injection was evaluated for NF-κB p65 DNA binding with ELISA. **c** Total NF-κB p65 protein in brain tissue at 3 h post-injection was evaluated by western blot and normalized to GAPDH. **d** Representative image of NF-κB p65 and GAPDH western blot bands used to calculate the total NF-κB p65 brain protein. Values are reported as mean percent of saline control ± SEM. An asterisk indicates a significant age effect (P < 0.05) and a dagger indicates a significant difference between mouse strains (*P* < 0.05). *n* = 3

NF-κB p50^−/−^ mice are missing the prototypical pro-inflammatory transcription factor NF-κB p50/p65, which is traditionally associated with the induction of many pro-inflammatory genes, including TNFα and IL-1β [34]. As such, the activation and expression characteristics of NF-κB p65 were tested to begin to explore the signaling necessary for the initiation and amplification of pro-inflammatory factors in the absence of NF-κB p50. Young adult and middle-aged NF-κB p50^+/+^ and NF-κB p50^−/−^ mice were injected with LPS and either whole brain total protein or nuclear extract was collected at 3 h post-injection. Analysis of NF-κB p65 DNA binding in nuclear extract found no differences in basal NF-κB p65 activation for any group tested in saline-treated mice (Fig. 7a, *p* > 0.05). As expected, LPS injection resulted in a significant increase in brain NF-κB p65 activation in both adult and middle-aged NF-κB p50^+/+^ mice, but NF-κB p50^−/−^ mice failed to show any increase NF-κB p65 activity in response to LPS (Fig. 7b, *P* < 0.05). Further, western blot analysis of whole brain protein extract showed a significant decrease in NF-κB p65 levels in middle-aged mice, regardless of genotype (Fig. 7c, *P* < 0.05), suggesting that drastic age-related and synergistic increases in microglial priming may occur through signaling independent of the prototypical NF-κB p50/p65 transcription factor.

## Discussion

Microglia in the aging brain display a primed phenotype characterized by increased basal pro-inflammatory cytokine expression, heightened reactivity to pro-inflammatory stimuli, and resistance to regulation, which has been implicated in the vulnerability of the aged brain to neurodegenerative diseases [12, 13]. However, the molecular mechanisms driving microglia to a primed and dysregulated phenotype in the aging brain are unclear. Previous reports implicate loss of NF-κB p50 function due to disease [32], ROS [7], aging [40, 42], and genetic deletion [7, 31], as a potential mechanism that could prime the microglial pro-inflammatory response [7, 31]. Here, we address the role of NF-κB p50 in regulating microglia in the aging brain and reveal that (1) NF-κB p50 activity in response to a pro-inflammatory triggers changes across the lifespan, which decreases in older mice; (2) aging synergistically amplifies pro-inflammatory priming due to loss of NF-κB p50 function in microglia and the brain in middle-aged mice; and (3) NF-κB p65 expression is reduced in the middle-aged brain and NF-κB p65 activity in response to LPS decreases in the middle-aged NF-κB p50^−/−^ brain, despite the presence of exaggerated neuroinflammation.

NF-κB p50 is an important redox-sensitive regulator of the microglial activation response, where oxidative modification, including the free radical form of NF-κB p50, has been associated with loss of NF-κB p50 function, augmentation/priming of the late-stage pro-inflammatory response, and vulnerability to chronic inflammation in microglia and the brain [7]. While the NF-κB p50/p65 heterodimer is a prototypical activator of the pro-inflammatory response in many myeloid cells including microglia, the NF-κB p50/p50 homodimer is a key repressor of the pro-inflammatory response [49]. Recent studies using NF-κB p50^−/−^ mice indicate that NF-κB p50 is sufficient but not necessary to induce microglial activation and neuroinflammation [7, 31]. Rather, the removal of pro-inflammatory inhibition that allows the elevated and chronic neuroinflammation has been implicated as the critical consequence of loss of NF-κB p50 for pro-inflammatory function of key pro-inflammatory factors in the brain [7, 31]. Importantly, the current study found higher levels of TNFα, IL-1β, and microglial activation in response to LPS in the brain of NF-κB p50^−/−^ mice for all ages investigated (Figs. 2, 3, 4, 5 and 6), as expected.

In the current study, we sought to discern how aging impacts NF-κB p50 in the brain. Consistent with the premise that oxidative stress increases in the aging brain [28], that ROS impairs NF-κB p50 function in microglia [7], and that aging in peripheral tissues is associated with a loss of NF-κB p50 function [40, 42], we found that 9-month-old mice exhibited less NF-κB p50 DNA binding in the brain when compared to younger 3-week-old mice (Fig. 1). While it should be noted that brain NF-κB p50 DNA binding in young mice may be affected by differences in developmental processes [37] and the multiple cell types in the brain, these age-specific effects in NF-κB p50 activity support that loss of NF-κB p50 function may occur in the brain with aging, which could contribute to the dysregulated pro-inflammatory phenotype in the aging brain.

We next sought to reveal how aging and loss of NF-κB p50 function interact to regulate the pro-inflammatory environment in the brain. Intraperitoneal LPS injections result in elevated circulating cytokines, such as TNFα, that cross the blood-brain barrier to activate microglia and cause neuroinflammation [44], emphasizing the importance of communication between the peripheral and CNS immune systems for brain health [26]. Here, we demonstrate that while loss of NF-κB p50 function causes robustly elevated circulating LPS-induced TNFα when compared to control mice, aging further augmented (× 6 amplification) this already primed response to a striking magnitude ($\overline{x}$ = 11,297 pg/mL TNFα). While the excessive priming occurred as early as 8.0–11.0 months of age and continued to 16.0–18.0 months of age with the serum (Fig. 2a), this type of priming was only seen at the later ages (16.0–18.0 months) in the brains of the NF-κB p50^−/−^ mice (Fig. 2c), suggesting that the brain’s vulnerability to the synergistic peripheral pro-inflammatory response increases with age.

Consistent with our prior report in young adult mice [7], loss of NF-κ B p50 function did not impact all pro-inflammatory mediators and preferentially augmented TNFα and IL-1β at 3 h in response to intraperitoneal injection of LPS at all ages explored. Interestingly, aging synergistically amplified the neuroinflammation priming induced by loss of NF-κB p50 function as seen in the midbrain (TNFα and IL-1β, Figs. 2c and 3b), isolated adult microglia (TNFα and IL-1β, Fig. 6a and b), and changed in microglia morphology in the substantia nigra (Figs. 4 and 5). Thus, data indicate that aging and loss of NF-κB p50 function interact to synergistically augment LPS-induced peripheral inflammation, neuroinflammation, and more specifically, the microglial pro-inflammatory response. However, these findings are unable to differentiate how much of the amplified neuroinflammation is due to the augmented peripheral pro-inflammatory response versus the sensitivity of the microglia to a pro-inflammatory trigger.

To further characterize the role of NF-κB in this age-induced synergistic response in the brain, we began by investigating NF-κB p65 activity and expression in the brain. Functional NF-κB p65 is present in NF-κB p50^−/−^ mice and has been previously implicated in combination with other transcription factors to participate in activation of pro-inflammatory genes [50], making NF-κB p65 the most likely candidate for initiation and amplification of neuroinflammation with age. Surprisingly, NF-κB p65 total protein expression was lowered in the brains of middle-aged mice, regardless of genotype (Fig. 7). Further, while NF-κB p50^+/+^ mice exhibited an LPS-induced increase in brain NF-κB p65 DNA binding in both young and aging mice, the NF-κB p50^−/−^ mice revealed a decrease in LPS-induced NF-κB p65 DNA binding, regardless of age (Fig. 7a and b). As such, these findings indicate a break between age-induced microglial priming in the brain and prototypical pro-inflammatory signaling that supports the presence of an unknown yet highly efficacious activator of pro-inflammatory genes with the absence or decline of NF-κB p50 and NF-κB p65.

## Conclusions

In summary, increasing reports indicate that loss of NF-κB p50 function in the brain may occur due to neurodegenerative disease [32] and ROS [7] and that brain NF-κB p50 activity in response to pro-inflammatory stimuli changes across a lifetime (Fig. 1). Middle age is an important time point in the life span to consider when exploring the potential mechanisms that may cause a pre-disposition to neurodegenerative diseases. In the current study, we reveal that aging (middle age) and loss of NF-κB p50 function interact to result in elevated basal neuroinflammation and synergistically augment microglial activation/neuroinflammation in response to a peripheral pro-inflammatory trigger, such as LPS. This is important because not only does it provide a possible rationale for the pro-inflammatory baseline in the aging brain, but it may also offer insight as to why the aging brain, even at middle age, may have a heightened response to disease and pro-inflammatory stimuli. The current study presents the hypothesis that it may be the additional loss of NF-κB p50 function across the lifespan that may be driving some of the age-specific vulnerability to pro-inflammatory stimuli in the brain. In fact, data reveal that a loss of function of both components of the prototypical pro-inflammatory transcription factor (NF-κB p50/p65) is associated with aging in the brain and that this occurs concurrently with the augmented pro-inflammatory response, pointing to a novel, unknown, and robust mechanism responsible for aging-induced neuroinflammation priming in middle-aged mice. As such, it remains of significant importance that future scientific inquiry focus on elucidating this unique deleterious signaling in an effort to identify potential markers and therapeutic targets capable of modifying the outcome of aging-related CNS diseases.

## Abbreviations

CD11b: Integrin alpha M and macrophage-1 antigen (MAC-1) alpha subunit
CD200: Cluster of Differentiation 200
CNS: Central nervous system
COX-2: Cyclooxygenase 2
DNA: Deoxyribonucleic acid
ELISA: Enzyme-linked immunosorbent assay
GAPDH: Glyceraldehyde 3-phosphate dehydrogenase
IL-10: Interleukin-10
IL-1β: Interleukin-1 beta
IL-4: Interleukin-4
IL-6: Interleukin-6
iNOS: Inducible nitric oxide synthase
LPS: Lipopolysaccharide
NF-κB: Nuclear factor kappa-light-chain-enhancer of activated B cells
RNA: Ribonucleic acid
ROS: Reactive oxygen species
TNFα: Tumor necrosis factor alpha

## References

[1] Hickman SE, Kingery ND, Ohsumi TK, Borowsky ML, Wang LC, Means TK, El Khoury J (2013). The microglial sensome revealed by direct RNA sequencing. Nat Neurosci.

[2] Sun Q, Wu W, Hu YC, Li H, Zhang D, Li S, Li W, Li WD, Ma B, Zhu JH (2014). Early release of high-mobility group box 1 (HMGB1) from neurons in experimental subarachnoid hemorrhage in vivo and in vitro. J Neuroinflammation.

[3] Rosenberger K, Derkow K, Dembny P, Kruger C, Schott E, Lehnardt S (2014). The impact of single and pairwise toll-like receptor activation on neuroinflammation and neurodegeneration. J Neuroinflammation.

[4] Aguzzi A, Barres BA, Bennett ML (2013). Microglia: scapegoat, saboteur, or something else?. Science.

[5] Block ML, Zecca L, Hong JS (2007). Microglia-mediated neurotoxicity: uncovering the molecular mechanisms. Nat Rev Neurosci.

[6] Heneka MT, Kummer MP, Latz E (2014). Innate immune activation in neurodegenerative disease. Nat Rev Immunol.

[7] Taetzsch T, Levesque S, McGraw C, Brookins S, Luqa R, Bonini MG, Mason RP, Oh U, Block ML (2015). Redox regulation of NF-kappaB p50 and M1 polarization in microglia. Glia.

[8] Perry VH, Holmes C (2014). Microglial priming in neurodegenerative disease. Nat Rev Neurol.

[9] McGeer PL, Itagaki S, Boyes BE, McGeer EG (1988). Reactive microglia are positive for HLA-DR in the substantia nigra of Parkinson’s and Alzheimer’s disease brains. Neurology.

[10] Pereira MD, Ksiazek K, Menezes R (2012). Oxidative stress in neurodegenerative diseases and ageing. Oxidative Med Cell Longev.

[11] Baron R, Babcock AA, Nemirovsky A, Finsen B, Monsonego A (2014). Accelerated microglial pathology is associated with Abeta plaques in mouse models of Alzheimer’s disease. Aging Cell.

[12] Norden DM, Godbout JP (2013). Review: microglia of the aged brain: primed to be activated and resistant to regulation. Neuropathol Appl Neurobiol.

[13] Niraula A, Sheridan JF, Godbout JP (2017). Microglia priming with aging and stress. Neuropsychopharmacology.

[14] Schuitemaker A, van der Doef TF, Boellaard R, van der Flier WM, Yaqub M, Windhorst AD, Barkhof F, Jonker C, Kloet RW, Lammertsma AA (2012). Microglial activation in healthy aging. Neurobiol Aging.

[15] Bardou I, Kaercher RM, Brothers HM, Hopp SC, Royer S, Wenk GL (2014). Age and duration of inflammatory environment differentially affect the neuroimmune response and catecholaminergic neurons in the midbrain and brainstem. Neurobiol Aging.

[16] Henry CJ, Huang Y, Wynne AM, Godbout JP (2009). Peripheral lipopolysaccharide (LPS) challenge promotes microglial hyperactivity in aged mice that is associated with exaggerated induction of both pro-inflammatory IL-1beta and anti-inflammatory IL-10 cytokines. Brain Behav Immun.

[17] Godbout JP, Chen J, Abraham J, Richwine AF, Berg BM, Kelley KW, Johnson RW (2005). Exaggerated neuroinflammation and sickness behavior in aged mice following activation of the peripheral innate immune system. FASEB J.

[18] von Bernhardi R, Tichauer JE, Eugenin J (2010). Aging-dependent changes of microglial cells and their relevance for neurodegenerative disorders. J Neurochem.

[19] Ye SM, Johnson RW (2001). An age-related decline in interleukin-10 may contribute to the increased expression of interleukin-6 in brain of aged mice. Neuroimmunomodulation.

[20] Maher FO, Nolan Y, Lynch MA (2005). Downregulation of IL-4-induced signalling in hippocampus contributes to deficits in LTP in the aged rat. Neurobiol Aging.

[21] Fenn AM, Henry CJ, Huang Y, Dugan A, Godbout JP (2012). Lipopolysaccharide-induced interleukin (IL)-4 receptor-alpha expression and corresponding sensitivity to the M2 promoting effects of IL-4 are impaired in microglia of aged mice. Brain Behav Immun.

[22] Wynne AM, Henry CJ, Huang Y, Cleland A, Godbout JP (2010). Protracted downregulation of CX3CR1 on microglia of aged mice after lipopolysaccharide challenge. Brain Behav Immun.

[23] Lyons A, Lynch AM, Downer EJ, Hanley R, O’Sullivan JB, Smith A, Lynch MA (2009). Fractalkine-induced activation of the phosphatidylinositol-3 kinase pathway attentuates microglial activation in vivo and in vitro. J Neurochem.

[24] Frank MG, Barrientos RM, Biedenkapp JC, Rudy JW, Watkins LR, Maier SF (2006). mRNA up-regulation of MHC II and pivotal pro-inflammatory genes in normal brain aging. Neurobiol Aging.

[25] Raj DD, Jaarsma D, Holtman IR, Olah M, Ferreira FM, Schaafsma W, Brouwer N, Meijer MM, de Waard MC, van der Pluijm I (2014). Priming of microglia in a DNA-repair deficient model of accelerated aging. Neurobiol Aging.

[26] Perry VH, Teeling J (2013). Microglia and macrophages of the central nervous system: the contribution of microglia priming and systemic inflammation to chronic neurodegeneration. Semin Immunopathol.

[27] Cahill-Smith S, Li JM (2014). Oxidative stress, redox signalling and endothelial dysfunction in ageing-related neurodegenerative diseases: a role of NADPH oxidase 2. Br J Clin Pharmacol.

[28] Droge W, Schipper HM (2007). Oxidative stress and aberrant signaling in aging and cognitive decline. Aging Cell.

[29] Qin L, Liu Y, Hong JS, Crews FT (2013). NADPH oxidase and aging drive microglial activation, oxidative stress, and dopaminergic neurodegeneration following systemic LPS administration. Glia.

[30] Cartwright T, Perkins ND, Wilson CL (2016). NFKB1: a suppressor of inflammation, ageing and cancer. FEBS J.

[31] Rolova T, Puli L, Magga J, Dhungana H, Kanninen K, Wojciehowski S, Salminen A, Tanila H, Koistinaho J, Malm T (2014). Complex regulation of acute and chronic neuroinflammatory responses in mouse models deficient for nuclear factor kappa B p50 subunit. Neurobiol Dis.

[32] Saldana M, Mullol J, Aguilar E, Bonastre M, Marin C (2007). Nuclear factor kappa-B p50 and p65 subunits expression in dementia with Lewy bodies. Neuropathol Appl Neurobiol.

[33] Oeckinghaus A, Hayden MS, Ghosh S (2011). Crosstalk in NF-kappaB signaling pathways. Nat Immunol.

[34] Vallabhapurapu S, Karin M (2009). Regulation and function of NF-kappaB transcription factors in the immune system. Annu Rev Immunol.

[35] Porta C, Rimoldi M, Raes G, Brys L, Ghezzi P, Di Liberto D, Dieli F, Ghisletti S, Natoli G, De Baetselier P (2009). Tolerance and M2 (alternative) macrophage polarization are related processes orchestrated by p50 nuclear factor kappaB. Proc Natl Acad Sci U S A.

[36] Baltimore D (2011). NF-kappaB is 25. Nat Immunol.

[37] Kaltschmidt B, Kaltschmidt C (2009). NF-kappaB in the nervous system. Cold Spring Harb Perspect Biol.

[38] Rolova T, Dhungana H, Korhonen P, Valonen P, Kolosowska N, Konttinen H, Kanninen K, Tanila H, Malm T, Koistinaho J (2016). Deletion of nuclear factor kappa B p50 subunit decreases inflammatory response and mildly protects neurons from transient forebrain ischemia-induced damage. Aging Dis.

[39] Jurk D, Wilson C, Passos JF, Oakley F, Correia-Melo C, Greaves L, Saretzki G, Fox C, Lawless C, Anderson R (2014). Chronic inflammation induces telomere dysfunction and accelerates ageing in mice. Nat Commun.

[40] Bernal GM, Wahlstrom JS, Crawley CD, Cahill KE, Pytel P, Liang H, Kang S, Weichselbaum RR, Yamini B (2014). Loss of Nfkb1 leads to early onset aging. Aging (Albany NY).

[41] Low JT, Hughes P, Lin A, Siebenlist U, Jain R, Yaprianto K, Gray DH, Gerondakis S, Strasser A, O’Reilly LA (2016). Impact of loss of NF-kappaB1, NF-kappaB2 or c-REL on SLE-like autoimmune disease and lymphadenopathy in Fas(lpr/lpr) mutant mice. Immunol Cell Biol.

[42] Yamini B (2015). Nfkb1/p50 and mammalian aging. Oncotarget.

[43] Sha WC, Liou HC, Tuomanen EI, Baltimore D (1995). Targeted disruption of the p50 subunit of NF-kappa B leads to multifocal defects in immune responses. Cell.

[44] Qin L, Wu X, Block ML, Liu Y, Breese GR, Hong JS, Knapp DJ, Crews FT (2007). Systemic LPS causes chronic neuroinflammation and progressive neurodegeneration. Glia.

[45] Harms AS, Tansey MG (2013). Isolation of murine postnatal brain microglia for phenotypic characterization using magnetic cell separation technology. Methods Mol Biol.

[46] Hutson CB, Lazo CR, Mortazavi F, Giza CC, Hovda D, Chesselet MF (2011). Traumatic brain injury in adult rats causes progressive nigrostriatal dopaminergic cell loss and enhanced vulnerability to the pesticide paraquat. J Neurotrauma.

[47] Wang S, Yan JY, Lo YK, Carvey PM, Ling Z (2009). Dopaminergic and serotoninergic deficiencies in young adult rats prenatally exposed to the bacterial lipopolysaccharide. Brain Res.

[48] Ling Z, Zhu Y, Tong C, Snyder JA, Lipton JW, Carvey PM (2006). Progressive dopamine neuron loss following supra-nigral lipopolysaccharide (LPS) infusion into rats exposed to LPS prenatally. Exp Neurol.

[49] Pereira SG, Oakley F (2008). Nuclear factor-kappaB1: regulation and function. Int J Biochem Cell Biol.

[50] Chen LF, Greene WC (2004). Shaping the nuclear action of NF-kappaB. Nat Rev Mol Cell Biol.

